# Limitations of transmucosal ketamine administration for chronic pain in dogs with osteoarthritis

**DOI:** 10.3389/fvets.2026.1934484

**Published:** 2026-10-29

**Authors:** Rowan Alaina Seabolt, Lindsay Hochman Elam, Gregg Mitchell Griffenhagen, Felix Michael Duerr

**Affiliations:** Department of Clinical Sciences, Colorado State University, Fort Collins, CO, United States

**Keywords:** absorption, canine, ketamine, mobility, osteoarthritis, pain, transmucosal

## Abstract

**Introduction:**

This prospective, open-label clinical trial aimed to evaluate the efficacy of low-dose keta-mine (0.5 mg/kg) administered by oral transmucosal route every 8 h in client-owned dogs with refractory, chronic osteoarthritis pain.

**Methods:**

Objective gait analysis was the primary outcome measure for orthopedic function relating to pain. Liquid chromatography-tandem mass spectrometry was used to quantify plasma ketamine and norketamine concentrations 30 and 60 min after administration of an oral transmucosal ketamine (TMK) gelatin lozenge.

**Results:**

Enrollment was discontinued early due to difficulty of administering TMK in the formulation provided (administration success = 54%; 181/334 doses in *n* = 9 participants). The result-ing sample size was too small for the intended statistical analysis of objective outcome da-ta to determine treatment efficacy. Mean plasma concentrations of ketamine and norketa-mine (*n* = 8 patients) were 4.5 ng/mL (+/− 3.0) and 47 ng/mL (+/− 38.3) at 30 min, and 1.7 ng/mL (+/− 1.2) and 65.7 ng/mL (+/− 46.7) at 60 min post administration.

**Discussion:**

These values suggest that TMK is absorbed and subsequently metabolized into norketa-mine. However, the low success rate of administration demonstrated that an oral transmu-cosal gelatin lozenge is an impractical delivery method for low-dose ketamine in a clinical setting. Evaluation of alternative TMK formulations and further research with an appropriate sample size is recommended to evaluate efficacy, pharmacokinetics, and pharmacody-namics before TMK can be used clinically.

## 1 Introduction

Osteoarthritis (OA) is the most common joint disease in human and veterinary patients, affecting both everyday mobility and overall quality of life (1), with prevalence of radiographically identifiable OA reported to be present in 40% of dogs aged 8-months to 4-years (2). Currently, there is no curative treatment for OA, and although there are surgical options which can be successful in select patients, the mainstays of treatment focus on symptom-modification prioritizing pain-control (3). Pharmaceutical options for pain control in dogs are limited, even more so when medication selection is constrained by comorbidities and organ dysfunction. When patients experience arthritis pain that is refractory to treatment, clinicians are forced to explore medications outside their established prescribing repertoire and are encouraged to work from the most evidence-supported to the least when exploring adjunct therapies or off-label applications of existing medications (4).

Ketamine is an emerging agent in the treatment of chronic pain for humans and animals. The drug is typically used for anesthesia and analgesia in surgical settings, with unique value attributed to its minimal depression of the cardiovascular or respiratory system (5). Ketamine has an existing FDA approval for use intravenously (IV) or intramuscularly (IM) for anesthesia and analgesia, and is classified as a dissociative anesthetic when administered at higher doses (6). Ketamine acts on many receptor types, including some opioid receptors, though its rising popularity in clinical studies is focused on its action as a non-competitive N-methyl-D-aspartate (NMDA) antagonist. Specifically, ketamine binds within the trans-membrane ion-channel of the NMDA receptor at the phencyclidine binding site and partially obstructs the magnesium binding site. While bound, the channel is non-competitively blocked, and delays influx of calcium, sodium, and potassium ions. There is an immediate impact from this delayed cation influx, resulting in activation of nitric oxide synthase, and a chronic impact from long-term potentiation of the NMDA receptor, where it is thought to impact neuroplasticity; this is where chronic pain can impact central sensitization (7). Additionally, ketamine has been found to bind to each opioid receptor (mu, kappa, and delta) with variable affinity, though this action is thought to have little contribution to the analgesic effects of ketamine (5).

Emerging studies in humans and animals are particularly focused on a variety of off-label doses, routes, and general treatment indications, such as the promising outcomes for human patients with unipolar and treatment-resistant depression (8, 9). Subanesthetic, or “low-dose” ketamine administration has been investigated in murine models and humans for relieving pain conditions (10), including diabetic neuropathy and opioid-resistant chronic pain (11). Chronic pain treatment protocols and doses in the veterinary field are currently based on anecdotal experience and only described in non-peer reviewed literature; the majority of which includes subcutaneous (SQ) doses or intravenous infusions at varying intervals (12). There have been some emerging studies on ketamine in canine patients, including a pharmacokinetic study on intravenous infusion in beagles (13), and a comparative bioavailability study of intranasal (IN) versus IV administration in beagles (14). While these sources contribute to the rising interest in clinical applications for ketamine, it also highlights the paucity of evidence from pharmacokinetic and efficacy standpoints which should guide potential treatment protocols, particularly in use of controlled substances. This is a significant concern in human patients, but also in responsible veterinary prescribing (15).

Clinical patients at the authors’ institution with severe, refractory, cancer-related pain have been managed with transmucosal ketamine (TMK) as a gelatin lozenge in addition to a multimodal pain management plan. This was initiated using a protocol developed at a different institution based on positive, though anecdotal, clinical experience with administration of 0.3–0.5 mg/kg every 6–8 h. While there is a lack of definitive, canine-specific data, this dose is supported by various studies through similar administration routes (10): a rodent model explored a single IN dose of 0.5 mg/kg for various types of facial pain (16), and two human studies described an oral dose of 0.5 mg/kg oral ketamine at intervals of 12 h for cancer pain (17) and every 8 h for neuropathic cancer-related pain, respectively (18). Clinical suggestions for treatment of dogs with refractory chronic pain suggest a dose of 0.5 mg/kg subcutaneously (12). A canine study utilizing 2 mg/kg IN ketamine reported no systemic adverse events (14). The dosing interval of 6–8 h appeared to be safe based on the total daily dose and rapid elimination time described in previous studies (10, 12, 14). A goal of this study was to evaluate feasibility of administration and objectively evaluate efficacy of this recently adopted TMK product in the treatment of refractory pain related to OA in canine patients.

This prospective, open-label, clinical trial aimed to evaluate and quantify absorption of TMK, and to assess response in canine patients receiving a multimodal treatment protocol for chronic pain attributed to OA. We hypothesized that TMK would be successfully absorbed and improve patient function quantified by statistically significant improvement in percent body weight distribution in kinetic gait analysis, and that effect on patient comfort would be observed with clinically meaningful improvement in veterinary and client-specific assessments.

## 2 Materials and methods

The study population consisted of client-owned dogs seeking care at Colorado State University (CSU), James L. Voss Veterinary Teaching Hospital (VTH). The study protocol was approved by the CSU VTH Clinical Review Board (IACUC Protocol #5381 approved February 15th, 2024). To detect improvement in comfort and function in an open-label study, kinetic data in the form of percent body weight distribution (%BWD) was used as the primary outcome measure. To detect a clinically meaningful difference, 5% change with a significance of 0.05 was used to calculate sample size. Previous studies in this facility using this system internally detected a standard deviation (SD) of 1.14 (19), and a recent study on temporospatial parameters in healthy dogs on an equivalent pressure sensitive walkway had SDs ranging from 1.53–3.5 (19, 20). For a power of 90%, an SD of 1.5 was selected, which would require a population of 27 dogs. To account for attrition of up to *n* = 5 patients over the course of the study, a desired study enrollment of *n* = 32. During a preliminary review of data after the participation of *n* = 9 dogs, it was elected to discontinue study enrollment without this adequately powered sample size due to client feedback on difficulty of administration of the transmucosal gelatine lozenge.

### 2.1 Enrollment criteria

Patients were considered eligible for enrollment if they had OA in at least one appendicular joint confirmed by radiographic imaging within the last 12 months. Enrolled patients had to display detectable mobility impairment such as lameness, or reduction in physical capabilities due to pain associated with arthritis. Lameness was subjectively identified on visual assessment by a veterinarian using a numeric 5-point lameness scale in which 0/5 is no visible lameness and 5/5 is a non-weightbearing lameness (21). Additionally, objective gait analysis was performed via a pressure-sensitive walkway with %BWD to quantify lameness via asymmetry of patient gait (22). Impacts of pain and lameness were evaluated using the pain severity score and pain interference score of the Canine Brief Pain Inventory (CBPI). Dogs were eligible for enrollment if their function was scored as ≥2 out of 4 in either category (23). Enrollees had to be in otherwise good health (i.e., free from systemic illness, or existing conditions must be well-controlled, without impact on patients’ quality of life, mobility, or activity levels as determined by the attending veterinarian at the time of enrollment). Enrolled patients were required to have an established and consistent pain management plan, including treatment with an NSAID on a daily basis.

### 2.2 Study design and timeline

Upon enrollment, dogs participated in data collection for a total of 24-days (Figure 1). This included a 10-day baseline data collection period, followed by a 2-week treatment period, with patients presenting for clinical evaluation on days 0, 10, 17, and 24. At each visit, the following outcome measures were collected: Objective gait analysis (OGA), physical and orthopedic examination by a veterinarian including Subjective orthopedic score (SOS) (24), client-specific outcome measures (CSOM), CBPI, and accelerometry data. Baseline data collection on day-0 and 10 (visits 1 and 2) allowed for evaluation of variability in baseline data, as well as allowing 10 days of activity collar data collection prior to initiation of treatment. After data collection on day 10, the first dose of TMK was administered in the hospital with plasma samples collected as described below for absorption analysis. While patients were in the treatment phase of data collection, clients also participated in daily surveys to report medication administration, any deviation from administration protocol (e.g., inadvertent chewing or swallowing of medication), and observed side effects. Post-treatment outcome measures were recorded on visit days 17 and 24 (visits 3 and 4) respectively.

**Figure 1:**
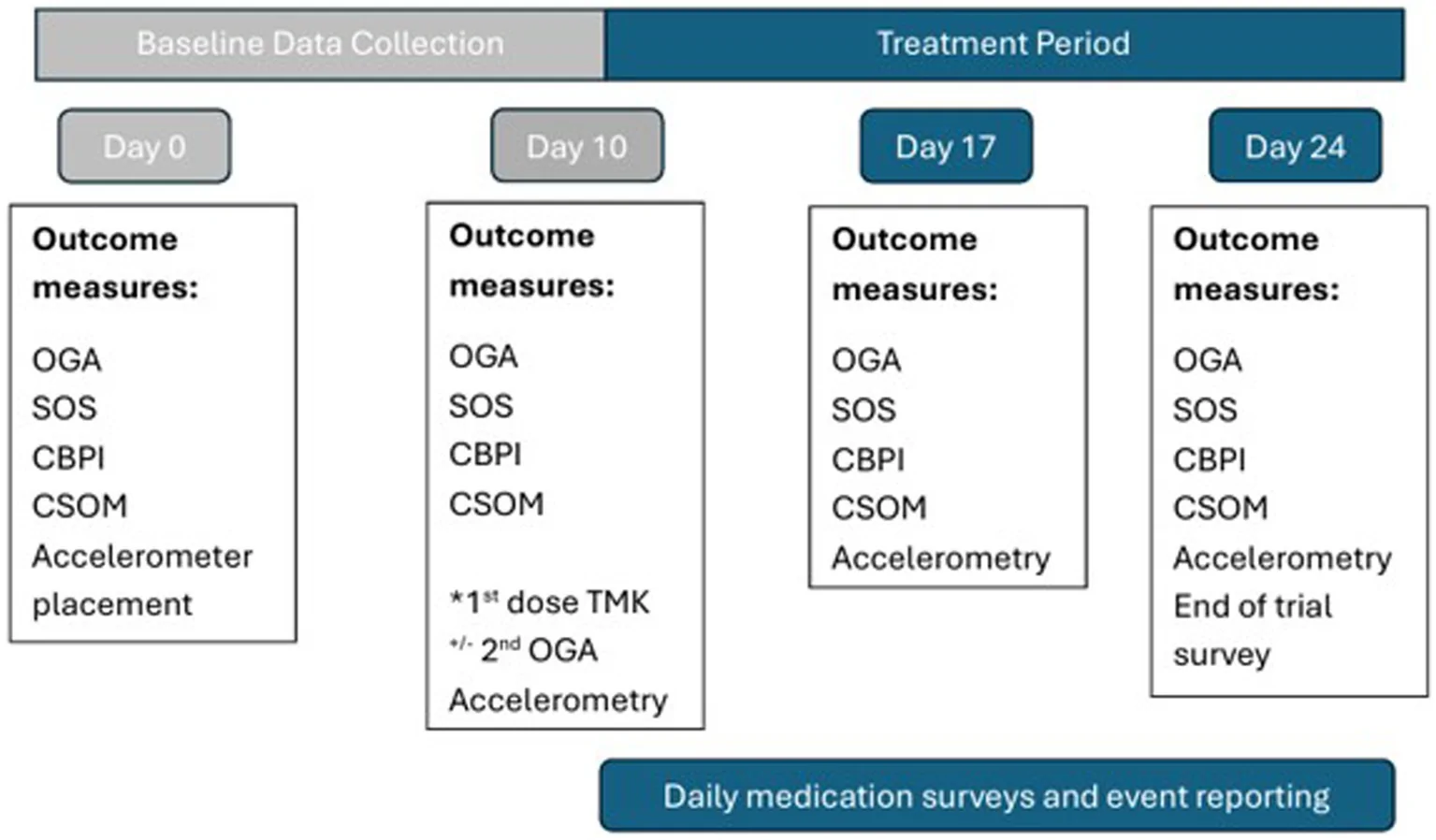
Study timeline illustrating visit dates from day 0, 10, 17, and 24 with a list of outcome measures performed at each visit.

### 2.3 Treatment

Study participants were administered a custom compounded prescription of ketamine in the form of a gelatin cube containing 0.5 mg/kg of ketamine per cube once every 8 h. The gel cube carrier was achieved using a compounding protocol for ketamine utilized by the CSU hospital pharmacy (Supplementary materials 7.1). The first dose of TMK was administered by clinical trials staff while collecting plasma samples for quantification of absorption on day 10 (visit 2). Clients were given written instructions with images and a verbal demonstration for medication administration. Clients were instructed to use gloves when handling medication, and to administer the TMK into the cheek pouch of their dog and hold the gel cube in place until it dissolved once every 8 h for the duration of the treatment period (Supplementary materials 7.2).

Clients were notified via the client consent form about the potential for abuse of ketamine by humans. Clients were asked to acknowledge the status of ketamine as a Schedule III drug, and consent to appropriate storage and handling, specifically in preventing accidental contact or absorption of the medication by anyone other than the prescribed dog.

### 2.4 Outcome measures

All study data (including client surveys) were collected and managed using REDCap (Research Electronic Data Capture) electronic data capture tools (Supplementary materials 7.3) (25, 26).

#### 2.4.1 Objective gait analysis

Objective gait analysis was collected at each visit with a pressure-sensitive walkway (6-Tile High Resolution Strideway System, Tekscan Inc., South Boston, Ma, United States) (27). Patients acclimated to the space prior to gait analysis at each visit, then data was collected using an established protocol (28). Patients were allowed to walk or trot at their preferred velocity, and subsequent gait analysis recordings were considered valid trials if the trials was within 0.3 m/s of the velocity of gait trials for that patient during the enrollment evaluation (visit 1) (22). Each patient was gaited until six trials of appropriate quality were achieved (in either direction), unless a patient was visibly painful or unable to perform further gaiting to accomplish 6 valid trials. On day 10 (visit 2), a second OGA was performed within an hour of administration of the first dose of TMK, unless clinical staff deemed a patient too painful to complete the additional distance of the repeated gait collections, to screen for any acute improvement in lameness in response to treatment.

#### 2.4.2 Accelerometry

Starting at the enrollment visit-1, dogs were fitted with an activity monitoring device (Actigraph wGT3X-BT; ActiGraphCorp, Pensacola, FL, United States) on a separate collar from any name tags or leash attachments with client instruction in appropriate monitor use (29). Monitoring of the device was continuous, with epoch length set to 60 s. At each subsequent visit, data was downloaded, and the collar was charged prior to replacement at visit discharge.

#### 2.4.3 Clinical metrology instruments

CMIs were administered throughout the study, which included the CBPI and CSOM documented at each visit. Clients were given the option to select from a list of activities and behaviors or supply their own to rate through the CSOM questionnaires as described in previous studies (19). Clients were instructed to choose a single caretaker to respond to surveys for the duration of the study to maintain consistency. If the chosen respondent was not present for a clinical visit, surveys were sent via email, or a printed sheet was provided for manual recording. If surveys were submitted in written form, they were transcribed into the digital survey by a clinical trials team member. Only complete surveys were included for analysis.

#### 2.4.4 Subjective orthopedic score

SOS was used to quantify exam findings by a veterinarian at each study visit, following a previously reported protocol (24). The SOS consists of 6 components intended to evaluate the level of pain and dysfunction. Each component was graded on a scale of 0–4, with 0 = normal and 4 = severe impairment (Supplementary materials 7.3). A total score sum was calculated for each visit, to compare pre- and post-treatment visit evaluations.

#### 2.4.5 Additional client surveys

In addition to CMI data, general patient data, daily medication logs, and client feedback were recorded through online surveys (Supplementary materials 7.3). General patient information including current medications and supplements was documented at the time of enrollment and updated at each visit. Between visit days 10–24, clients received daily surveys via email, with a daily medication log, and the option to submit an adverse event or side-effect report. The daily medication log included timing of medication administration, whether the pet chewed or swallowed any component of the gelatin lozenge, and room for any additional notes or comments.

An end of study survey was conducted, further assessing administration difficulty, client-perceived changes in demeanor and mobility, opinions on the product, including ease of administration on a scale of 0–10 (0 = very easy, 10 = nearly impossible), and intent to pursue ongoing treatment if commercially available (Supplementary materials 7.3).

### 2.5 Absorption and timed plasma analysis

Ketamine plasma analysis was modeled after previous ketamine efficacy studies (30–32). The authors estimated that initial plasma concentrations of ketamine and norketamine could be captured within 1-h of administration, for an adequate initial exploratory analysis. Therefore, samples were collected at time 0, just prior to administration of the first dose of TMK on day 10 (visit 2), and subsequently at 30 min and 60 min post-administration.

Three milliliters of blood was drawn from the jugular vein preferentially, or a peripheral vein in cases of patient discomfort during handling. Blood was immediately placed into lithium heparin tubes, gently mixed by inversion at least 5 times, and maintained in a refrigerator at 4 °C until sample processing (no more than 90 min after drawing the baseline sample at time 0). Samples were centrifuged for 15-min at 1534xg (3,500 rpm) using a commercially available centrifuge (LW Scientific Model E8 Portable Centrifuge, Lawrenceville, GA, United States). The plasma supernatant was collected with a single-use pipette into a microtube, labeled and stored at −80 °C until analysis could be performed.

#### 2.5.1 Plasma ketamine and norketamine analysis

Ketamine and norketamine plasma concentrations were analyzed from the stored plasma samples using a previously described protocol (33). Briefly, samples were analyzed with an AB Sciex 6,500 quadrupole mass spectrometer (SCIEX, Framingham, MA, United States) with initial liquid chromatography separation via a Shimadzu Nexera UHPLC system equipped with LC-30 AD solvent delivery units and LC-30-AD HPLC integrated autosampler (Shimadzu Corporation, Kyoto, Japan). Initial separation was accomplished using a Suplelco Discovery 5um HS C_18_ column (Sigma-Alderich, Saint Louis MO) with a liquid injection volume of 10ul and a flowrate of 0.4 mL/min. The solvent consistent of Acetonitrile/Acetic acid (9,1 vol,vol). The chromatographic program increased the mobile phase from 0–2% over 0.2 min, then to 45% by 2.5 min, reaching 75% at 3.7 min. The sample was maintained at 75% until 4.5 min, and then decreased in a linear fashion back to 2% by 5.2 min. For both ketamine and norketamine detection a 5.2-min method was utilized, and mass spectrometry analysis was performed with collision-energy of 34.6v. To quantify ketamine, a Q1 excitation wavelength of 238–125 and 238-179 nm was utilized. For norketamine quantification, a Q1 excitation wavelength of 224-207 nm and 224-179 nm was utilized. Values after quantification were rounded to the nearest 1000th of a decimal.

Working standard solutions were prepared by diluting ketamine and norketamine (1 mg/mL in methanol, Millipore Sigma, Saint Louis MO) in acetonitrile (ACN) to concentrations of 1–1,000 μg/mL. Calibrators were prepared by adding working standards to drug free canine plasma to obtain concentrations of 0.1–100 μg/mL. Both plasma samples and calibrators were prepared for analysis by combining 250 μL of sample with 300 μL of solvent (Acetonitrile/Acetic acid (9,1 vol,vol)), vortexing, and chilling for 30 min at 4 °C. The sample was then centrifuged and the resulting supernatant harvested and placed into microcentrifuge vials. Fifty microliters of the supernatant were subsequently added to microsampling vials for analysis. The lower limit of quantification (LLOQ) of this system was 1 ng/mL; as such, concentrations below this threshold were set to 0 ng/mL, reflecting the assays calibration limits rather than a true presence of ketamine in the sample.

### 2.6 Statistical analysis

Due to early discontinuation of the trial and insufficient sample size (n = 9), formal analysis of treatment efficacy was not performed. Data on feasibility of dosing and client feedback is discussed in the format of a feasibility analysis of this smaller population, with discussion of barriers to clinical application and proposed updates for future studies. The available data from each outcome measure is presented with descriptive statistics where relevant. Serum analysis is described with raw data and simple descriptive statistics as a “proof of concept” for transmucosal absorption in canine patients.

## 3 Results

A total of 9 dogs participated in this clinical trial through the duration of baseline and treatment data collection. Of 9 enrolled dogs, all patients completed the required clinical visits and physical data collection, with 7/9 completing all study surveys. While the study was intended to investigate efficacy of treatment, the authors elected to discontinue data collection in response to the low successful administration rate and feedback that medication administration was exceedingly difficult for clients (see 3.3). This barrier to consistent administration was determined impractical by the authors and study enrollment was therefore discontinued.

The population consisted of 6 females, of which 5 were spayed, and 3 castrated males with patient age ranging from 4 to 14 years (mean 9.1 ± SD 3.3). Patient body weight ranged from 11.9 kg to 40.6 kg (mean 25 kg ± SD 9.5), and patient body condition score (BCS) ranged from 5/9 to 7/9 with mean patient BCS of 5/9 (SD ± 1).

### 3.1 Ketamine and norketamine plasma quantification

Plasma analysis is reported here for 8 of the 9 participating dogs for baseline (time 0). One patient was excluded from this analysis due to a deviation from protocol. Each patient had a baseline sample (time 0) collected prior to administration of the first dose of TMK. At time 0, the average ketamine plasma concentration registered as 0.36 ng/mL (SD 0.1) and norketamine registered as 0 ng/mL (SD 0) (equivalent to 0 ng/mL based on the calibrated LLOQ of the assay). Plasma ketamine 30-min post administration ranged from 1.83 to 9.8 ng/mL, with an average of 4.5 ng/mL (SD 3.00); and samples taken 60-min after administration ranged from 0.75 (equivalent to 0 ng/mL based on the LLOQ) to 4.19 ng/mL with an average of 1.7 ng/mL (SD 1.22). Plasma norketamine 30-min post administration ranged from 26.2-143 ng/mL with an average of 47 ng/mL (SD 38.28); and at 60-min post administration ranged from 14.4–148.0 ng/mL with average of 65.7 ng/mL (SD 46.69) (Figure 2; Table 1). Patient 9 experienced a deviation from protocol: The sample of whole blood from time 0 prior to administration was damaged after collection. A new whole blood sample was promptly collected 3-min post administration of the TMK, and the remainder of samples were collected per study protocol at 30 and 60-min post administration (Table 2).

**Figure 2:**
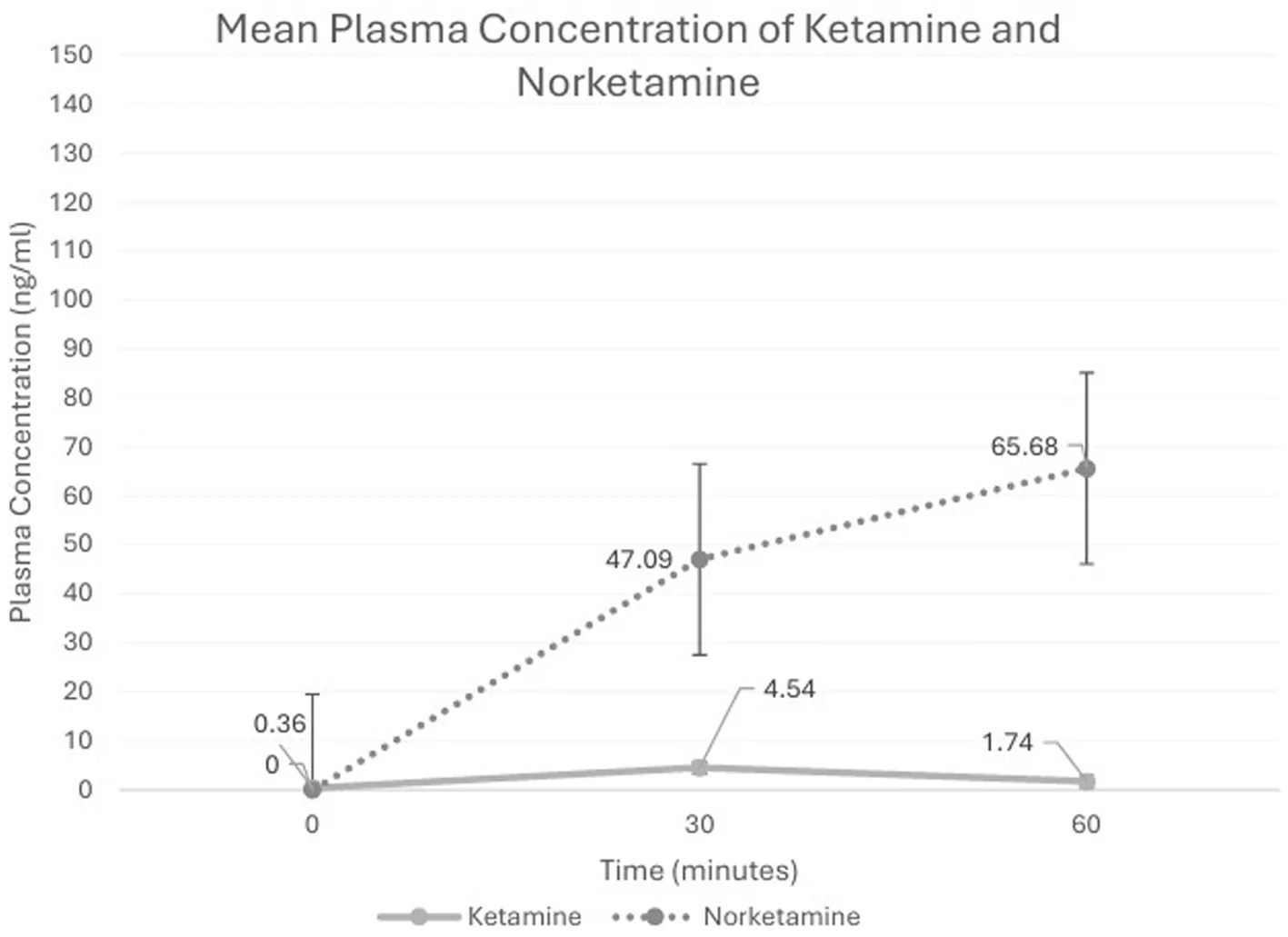
Mean values for plasma ketamine and plasma norketamine concentrations in ng/mL for each respective timepoint: prior to medication administration “time 0,” 30 min post administration, and 60 min post administration. The upper limit of plasma concentration demonstrates the desired range in ng/mL based on available literature for ketamine plasma concentrations.

**Table 1:** Plasma chromatography results.

Sample ID	Ketamine concentration (ng/mL)	Norketamine concentration (ng/mL)
A0	0.392	< 0
A30	2.9	143
A60	1.29	148
B0	0.378	< 0
B30	6.21	55.4
B60	2.67	86.4
C0	0.346	< 0
C30	6.46	44.6
C60	1.35	14.4
D0	0.368	< 0
D30	1.83	58.5
D60	0.75	71.7
E0	0.323	< 0
E30	3.01	35.3
E60	2.31	139
F0	0.651	< 0
F30	8.23	26.2
F60	1.23	19.1
G0	0.384	< 0
G30	2.39	29.6
G60	1.91	37
H0	0.409	< 0
H30	9.87	31.2
H60	4.19	75.5

Results of plasma liquid-chromatography. Patients are labeled alphabetically with “A” corresponding to patient #1 through “H” corresponding to patient #8. Data is listed in chronological order, with time 0 (immediately prior to administration), 30-min post administration, and 60-min post administration. Values ≤1.0 in this assay are equivalent to a concentration of 0 ng/mL based on the calibration of the LLOQ of the assay.

**Table 2:** Lists results of plasma liquid-chromatography for patient #9 labeled as “I” in this table had a deviation of protocol due to loss of the sample from time 0.

I3	69.8	44.6
I30	1.19	108
I60	0.499	102

A replacement sample was collected immediately after TMK administration, with clinical notes identifying the replacement sample was acquired approximately 3-min after TMK administration. Data is listed in chronological order, with time points at 3-min, 30-min, and 60-min post administration.

### 3.2 Outcomes in patient comfort and function: OGA, accelerometry, SOS, CMIs

Outcome measures including accelerometry collar data, OGA, clinician SOS, and CMIs including CBPI and CSOM were collected and evaluated. Due to the limited population size, there is minimal clinical significance of these values. Data is available for review in Supplementary materials 7.4.

### 3.3 Medication administration and additional client surveys

Each patient was dispensed a total of 42 doses for dosing every 8 h in the 2-week treatment period. For all 9 dogs, a total of 378 doses were compounded and dispensed, and of these doses 334 were documented in the daily administration surveys. Answers were reviewed individually and divided into successful transmucosal administration, obvious chewing or swallowing, other issues (spit out, dissolved etc.), or missed doses. Of the 334 documented doses, 181 (54%) were successful, 113 (34%) were chewed or swallowed, 30 (9%) experienced other complications such as spitting out or dissolving into the glove or onto the ground, and 10 (3%) missed doses were reported. A total of 44 doses had no documentation on whether they were administered successfully vs. experiencing complications (Supplementary materials 7.4).

To understand client perceptions of TMK in this open-label trial, an end-of-trial survey was distributed with 7/9 clients completing the responses. Clients were asked clients if they noted any change in their pet after using the product. Every respondent said “yes” with a subsequent free-text response section, where clients were then asked to elaborate. The majority of clients reported changes in patient energy level, with 3/7 reporting sedation or sleepiness, and 2/7 clients noted increased energy lasting 1–2 h after administration. The next prompt asked if they felt their pets’ mobility improved on this medication, where 5/7 clients answered “yes.” One client utilized the free response section to report improvements in rising from rest, and another reported markedly increased energy with a return of jumping into the car-noting it had been a year since that activity was observed. Of the 2/7 respondents who answered that there were no observable improvements in their pets’ mobility, both were the same clients who indicated sedation after administration.

The average score for ease of administration was 6 out of 10, indicating significant difficulty in client administration. When asked whether they would continue treatment if the product were commercially available, 57% (4/7) of respondents said no. A subsequent open-ended question asking to explain why they selected “yes” or “no” revealed that administration difficulty was the primary barrier. Even those willing to continue (3/7) preferred an easier delivery form or would reserve the current formulation for severe pain only (Supplementary materials 7.4).

## 4 Discussion

To the authors’ knowledge, this is the first clinical study to evaluate administration of oral transmucosal ketamine to canine patients. With the rapid increase in ketamine use through various formats in both human and veterinary medicine (6), objective data on the absorption, metabolism, efficacy, and administration feasibility of this medication is essential to guiding recommendations for best practices.

The results from this study indicate that while TMK as a gelatin lozenge appears to be absorbed and metabolized into norketamine, it is an impractical administration method for use in clinical practice. Plasma analysis was performed with a baseline sample just before the first dose at time 0, with subsequent 30-min and 60-min post administration sample collections. These paired plasma norketamine concentrations suggest that ketamine in the form of a gelatin lozenge is absorbed via transmucosal administration. The analysis also supports that subsequent metabolism into norketamine occurs over a similar duration of time when compared to existing literature on subanesthetic ketamine after it has reached peripheral circulation following IM, IV, IN, sublingual and oral administration (14, 32, 34). However, absorption of ketamine via transmucosal lozenge administration resulted in low concentrations of ketamine in the peripheral circulation ~4.5 ng/mL, in contrast to existing canine studies with IV administration where100-200 ng/mL is achieved within the first 5-min of administration (33). What was absorbed appeared to be rapidly metabolized into norketamine over the course of 1-h.

Compared to other clinical trials reporting plasma concentrations, the values with TMK administration in our study are low compared to IM or IV administration with a comparable dose. In human patients on low-dose ketamine therapy, 0.5 mg/kg of ketamine administered IM resulted in an acute reduction of pain threshold and plasma concentration average of 118 ng/mL (34). Other studies with human subjects suggest that a plasma concentration of 100-150 ng/mL is necessary to have a significant impact on hyperalgesia or allodynia (35). However, a recent pharmacokinetic study of low-dose ketamine in canine patients (13) suggests the timeframe for peak plasma concentrations of ketamine vs. norketamine is rapid, with IV ketamine reaching peak concentration around 150 ng/mL at 5-min post administration. This is further supported by a recent systematic review of peri-operative ketamine in feline and canine patients which identified a target plasma concentration of >200 ng/mL to achieve changes in the nociceptive threshold (36). By 30-min post IV administration in these patients, the average concentration of ketamine was less than 50 ng/mL. The authors suspect a rapid peak of plasma ketamine could have occurred between 0- and 30-min and began to decline as metabolism into norketamine occurred. This is supported by the data available from patient #9, where a deviation in protocol occurred, resulting in a baseline plasma sample collected 3-min after administration, rather than a pre-administration collection at time 0. This showed that 3-min post administration, plasma ketamine was much higher than any other sample (69.8 ng/mL) and already in the process of rapid metabolism into norketamine (44.6 ng/mL). This higher concentration of plasma ketamine and norketamine suggests that following transmucosal ketamine administration a peak plasma concentration may occur and begin to diminish within the first 5–10 min of administration, similar to IV or IM administration, though formal pharmacokinetic analysis with a larger patient population would be required to confirm this suspicion. Future studies aiming to fully characterize pharmacokinetics and bioavailability of ketamine should consider a design with more frequent sampling in the scale of 1 to 5-min intervals as performed in recent pharmacokinetic studies (13, 14).

While this study established basic proof of absorption of ketamine through transmucosal administration, the sample size is too small to determine if it is absorbed in a sufficient dose to provide relief from chronic pain states. Review of available literature on ketamine in low-dose administration demonstrates a substantial gap in pharmacokinetic data in our canine patients, with variable recommendations for target plasma concentration to achieve antinociceptive effects (14). The data for plasma concentration was collected after the initial administration in hospital by trained veterinary professionals who were able to ensure the gelatin remained within the buccal pouch until it was completely dissolved. However, one of the most significant barriers for clients was the patient chewing and swallowing the product, resulting in oral ingestion. It is well documented that ketamine has poor oral bioavailability in humans and animals due to first-pass effect and rapid metabolism by the liver.

Ketamine is a Schedule III controlled substance, an assignment by the United States Drug Enforcement Administration (DEA), used to classify a drug’s medical use. As a Schedule III drug, ketamine is considered to have high risk for abuse and the possibility of leading to either psychological or physical dependence by humans (37). Given the risks for abuse or possible drug diversion, emerging studies on ketamine should not only evaluate efficacy but quantify its merit over other available medications. For example, NMDA antagonists such as amantadine are readily available and a non-Scheduled drug by DEA classification (38). Future studies evaluating ketamine should consider placebo-controlled, or positive-controlled studies (for example utilizing amantadine) to help differentiate the benefits and risks of each NMDA antagonist on chronic pain management.

This open-label clinical study was intended to address both efficacy in transmucosal absorption, as well as efficacy in treatment of chronic pain related to OA. While the goal of this trial was to evaluate the efficacy and merit of subanesthetic ketamine in a multimodal protocol combined with an NSAID, the administration difficulty proved to be an unforeseen barrier to this treatment protocol. Ease of administration plays a significant role in treatment feasibility since it interferes with treatment compliance, particularly with veterinary patients. If the clients are unable or unwilling to give a medication consistently, then the overall treatment efficacy will be reduced, even in a functional product (39). The transmucosal gelatin lozenge was utilized in this clinical study based on favorable, but subjective preliminary clinical impression. In practice, this gelatin lozenge retained its shape and structure for a prolonged period while held in the buccal pouch, delaying absorption and allowing patients to chew or spit out the product and interfering with successful administration. The observed discrepancy to this study’s findings highlights the gap between subjective impressions derived from clinical feedback and findings generated through structured research methodology. Researchers should consider evaluations of other routes of administration or a reformulation of a transmucosal product to allow more rapid absorption for future studies, such as that of buccal oromucosal (transmucosal) dexmedetomidine gel for dogs, which was found to have high owner-reported usability (40).

While clients of this study reported visible changes in their pet such as improved energy level or visible “sleepiness” after administration in the end-of-trial survey, it is important to remember the potential impacts of caregiver placebo in this open-label study design (41). Sample size for this population was too small to extrapolate any clinical significance within the objective outcome measures for participants. The authors acknowledge the limitations of the reported benefits in this dataset. Some individual clients reported positive effects for their dogs in terms of energy and activity, but these same individuals gave negative feedback on the challenge of administration in the end of study survey. The authors note these juxtaposed results may warrant ongoing exploration of low-dose ketamine therapy through an alternative administration route or improved product for transmucosal administration.

Additionally, the impacts of norketamine as a sole agent on acute and chronic pain have only just begun to be explored, with suggestion of anti-nociceptive properties in a murine model (42). It is unclear at this time whether the focus of chronic pain treatment should be on achieving any particular plasma concentration of ketamine or norketamine for the desired outcomes for chronic pain relief, especially with limited availability of rigorous studies on norketamine in comparison to ketamine (35).

The authors note that there is a paucity of data available on pharmacokinetics, including bioavailability, half-life, and rate of elimination of ketamine administered through novel routes including oral, mucosal, nasal, and subcutaneous administration. This data is vital to guide decisions regarding dose, frequency and duration of treatment for future studies and possible application to clinical patients. The selection of the 0.5 mg/kg dose administered once every 8 h was ultimately based on anecdotal evidence. Future studies should review the emerging pharmacokinetic data and pharmacodynamic effects of ketamine in dogs to guide an evidence-based decision on dose, frequency, and duration of treatment.

This study was designed as an open label clinical trial, without a control group. Therefore, quantification of treatment success relied on objective measures including gait analysis and accelerometry data to remove the impact of caregiver placebo bias. Without a sufficient sample size, this objective data lacks the statistical power required to draw any conclusions on the clinical effect of ketamine on canine pain related to OA. The authors propose future studies should include a sufficient sample size, and ideally a double-blind placebo-controlled or even crossover design to evaluate true clinical impact of this treatment.

## 5 Conclusion

Our results suggest that there is some absorption of ketamine via transmucosal route, with a subsequent rapid metabolism to norketamine. An unforeseen barrier to treatment administration, and therefore any potential treatment impact, was the challenge clients reported in consistent administration of the gelatin lozenge formulation of the TMK to their dog. Consideration should be given to other carriers for the transmucosal administration route. Due to early discontinuation of study enrollment, resulting in a significantly smaller sample size than intended, this study was unable to objectively evaluate the effects of treatment with TMK on canine OA pain. Further research is required to understand the behavior of ketamine through the variety of routes and range of dosages currently recommended for the treatment of pain associated with OA.

## Data Availability

The datasets presented in this study can be found in online repositories. The names of the repository/repositories and accession number(s) can be found in the article/Supplementary material.
