# Supplementary material for: Limitations of transmucosal ketamine administration for chronic pain in dogs with osteoarthritis
Source: Front Vet Sci. 2026 Oct 29;13:1934484. doi: 10.3389/fvets.2026.1934484 (PMC13754547; doi:10.3389/fvets.2026.1934484)
Supplement: Supplementary file 1 [file Data_Sheet_1.PDF]

**Name of compounded product:** Ketamine Lozenges	**Protocol number and version:**
**Concentration:** As ordered (per 30 ct tray)	**Effective date:**
**Pharmaceutical form:** Lozenge	**Developed by:** SDD
**Route of administration:** Transmucosal	**Verified by:**

Formula

Ingredient	MFR	Quantity	Physical description	Additional information
Ketamine		Calculate	Clear Liquid	
Water		QS 30 ml	Clear Liquid	
NaCl		1.5 Grams	White Solid Granules	
Gelatin		3 Grams	Fine Powder	
Flavor		3 Drops	Colored Liquid	Beef or Peanut Butter

Notes on calculations and measurements, expected yield:

Make a batch of 30 ml of gelatin base, calculate the amount of ketamine used and subtract that from the 30 ml total volume to make 30 lozenges.

Required equipment, instruments and materials:

2 60 ml LL syringes
Scale
Syringe for ketamine
1 Leur Lock-to-Luer Lock RAPIDFILL Connector
1 30 count lozenge tray calibrated to 1 ml per lozenge

Compounding method:

Heat enough water in the microwave to measure out 30 ml
Measure out required ingredients
Combine the warm water, gelatin, NaCl, and flavor in a beaker and stir till dissolved.
In one 60 ml syringe draw amount of solution needed, in the second syringe draw amount of ketamine needed. (For smaller amounts use appropriately sized syringe)
Remove excess air from syringes, attach with Leur Lock-to-Luer Lock RAPIDFILL Connector.
Pass through connector until mixed.
Fill lozenge tray with 30 ml of final product
Let set for 1 hr (can set in fridge or on counter)

Quality Controls	Expected Specification
Visually inspect final volume	30 ml total volume
Visually inspect liquid	Fluid should be translucent and free flowing

Packaging:

Package in child proof lozenge tray with a label.

Stability and storage:

	Beyond-use date:	Storage conditions:
Store in the refrigerator.<br>*Medication tends to dry out with extended storage, limit to 30-day supply.<br>Storing in a plastic bag will help with drying in refrigerator.	90 days	Fridge

Labelling:

Store in refrigerator.
Wear Gloves
Keep out of reach of children.

References:

USP 795 (2024)

Guidance Document for Pharmacy Compounding of Non-sterile Preparations — Companion to the Model Standards for Pharmacy Compounding of Non- sterile Preparations, National Association of Pharmacy Regulatory Authorities' (NAPRA). Published March 2018. Revision June 2018

Approved 01/01/2024
