# Supplementary material for: Limitations of transmucosal ketamine administration for chronic pain in dogs with osteoarthritis
Source: Front Vet Sci. 2026 Oct 29;13:1934484. doi: 10.3389/fvets.2026.1934484 (PMC13754547; doi:10.3389/fvets.2026.1934484)
Supplement: Supplementary file 2 [file Data_Sheet_2.PDF]

TM Ketamine Dose Administration:

Transmucosal ketamine is designed to be absorbed through the skin within the mouth. Below are instructions for safe handling and ideal administration of transmucosal gel cubes.

1. **Wear Gloves**: please put on gloves prior to opening the container for the ketamine gel cubes.
2. Gently remove the gel cube from the dispensed container.
3. With your dog in a safe location, gently introduce one hand to the top of their head, as you would to pet them, and slide down to the corner of their mouth.
4. Insert 1 to 2 fingers in the corner of the mouth to get access to the cheek pouch, then place the gel cube into the deepest pocket of the cheek pouch before releasing the lips.
5. Monitor for 1 minute to ensure the cube is not spit out and take note if the dog appears to chew or swallow the gel instead.

   Example of how to hold the medication in the cheek pouch:

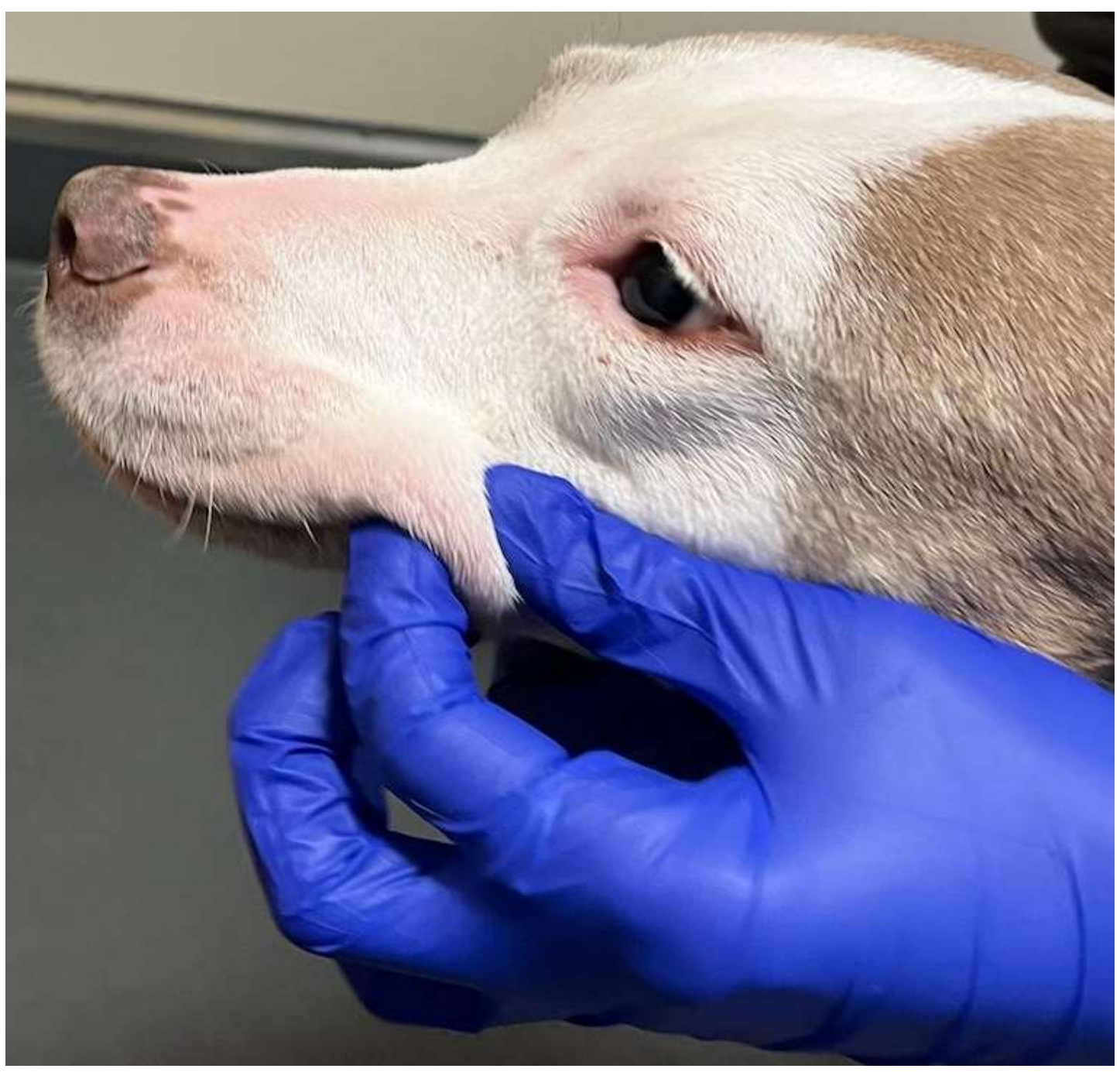

<u>**Ketamine Dose Log : EXAMPLE**</u>

Ketamine Dose Day #	**Date of administration:**	**Time of administration:**	**Did your pet chew or swallow the cube?**	**Additional notes:**
1				
2				
3				
4				
5				
6				
7				
