# Supplementary material for: Limitations of transmucosal ketamine administration for chronic pain in dogs with osteoarthritis
Source: Front Vet Sci. 2026 Oct 29;13:1934484. doi: 10.3389/fvets.2026.1934484 (PMC13754547; doi:10.3389/fvets.2026.1934484)
Supplement: Supplementary file 3 [file Data_Sheet_3.PDF]

Transmucosal Ketamine- Clinical Trial Patient Surveys

Introduction:

Provided is an example of each survey form provided to owners through the duration of the study. The study data was collected and managed using REDCap electronic data capture tools hosted at Colorado State University. REDCap (Research Electronic Data Capture) is a secure, web-based application designed to support data capture for research studies, providing: 1) an intuitive interface for validated data entry; 2) audit trails for tracking data manipulation and export procedures; 3) automated export procedures for seamless data downloads to common statistical packages; and 4) procedures for importing data from external sources. (1, 2)

Contents

Transmucosal Ketamine- Clinical Trial Patient Surveys 1

Introduction: 1

Adverse Event and Side-Effects Report: 2

Medications and Supplements: 5

End of Trial Survey: 6

References: 7

Adverse Event and Side-Effects Report:

Please complete the provided survey. After this survey, we will ask that you completed the "Medication and Supplements" survey.

The same person who fills out the initial survey has to be the same person to fill out the surveys for the remainder of the trial.

If you are unable to fill out these surveys using an electronic device, please notify us, and we can provide physical copies to be filled out.

Client Name __________ Patient Name ________ Date __________

Is [Patient Name] experiencing any general weakness?

☐ Yes ☐ No

[If yes:]
Please describe the general weakness you are seeing:

Enter free-response here

Has [Patient Name] skipped any meals?

☐ Yes ☐ No

[If yes:]
Please describe when a meal was skipped. Include information for frequency, amounts, and diet information.

Enter free-response here

Has [Patient Name] experienced any vomiting since last visit?

☐ Yes ☐ No

[If yes:]
Please describe how much and how often [Patient Name] has been vomiting. Is there a trend to when the vomiting occurs? What were the contents?

Enter free-response here

Has [Patient Name] experienced any diarrhea since last visit?

☐ Yes ☐ No

[If yes:]
Please specify how often [Patient name] has been experiencing diarrhea. Describe the consistency of the stool.

Enter free-response here

Has [Patient Name] experienced any coughing?

☐ Yes ☐ No

[If yes:]
Please specify how often [Patient name] has been coughing, Describe the sound or any trend for when coughing occurs.

Enter free-response here

Has [Patient Name] experienced any sneezing?

☐ Yes ☐ No

[If yes:]
Please describe how much and how often [Patient Name] has been sneezing. Does the sneeze produce any discharge? What does the discharge look like? Is there any trend to when the sneezing occurs?

Enter free-response here

Has [Patient Name] experienced increased frequency or volume of urination?

☐ Yes ☐ No

[If yes:]
Please describe how frequent [Patient Name] is urinating. When did the change in urination start? Has [Patient Name] been straining to urinate?

Enter free-response here

Has [Patient Name] experienced any increased thirst?

☐ Yes ☐ No

[If yes:]
Please describe how much [Patient Name] has been drinking. If you know specific measurements, please clarify. When did increased thirst begin?

Enter free-response here

Has [Patient Name] experiencing any increased appetite?

☐ Yes ☐ No

[If yes:]
Please describe [Patient Name]'s increased appetite.

Enter free-response here

Has [Patient Name] experienced any itching or skin irritation?

☐ Yes ☐ No

[If yes:]
Please describe how much and how often [Patient Name] has been itching. If irritation is present, where is it located and when did you notice it? Please specify any discoloration, inflammation, missing fur, crusting, and/or discharge. Is this similar or different to any existing seasonal or food allergies?

Enter free-response here

Has [Patient Name] experienced any worsened pain? An example would include non-weight-bearing on an affected limb, changes in behavior associated with mobility, etc.

☐ Yes ☐ No

[If yes:]
Please describe the pain [Patient Name] is experiencing. Have you noticed if it is centralized to a single location? Is it consistent, or is there a trend when this pain occurs? Have you witnessed any trauma or accidents?

Enter free-response here

Is [Patient Name] experiencing any other potential side-effects?

☐ Yes ☐ No

[If yes:]
Please describe in detail what [Patient Name] has been experiencing.

Enter free-response here

Medications and Supplements:

Patient Name_________ Date____________

Please list all medications and supplements that [Patient Name] is taking. Include dose, amount, and frequency.

End of Trial Survey:

Thank you for participating in our trial! Please complete the survey below to tell us how your experience was with this trial.

If you have any questions or concerns, please feel free to reach out to the study personnel. If you are unable to fill out these surveys using an electronic device, please notify us and we can provide physical copies to be filled out.

Thank you!

Orthopedic Medicine and Mobility – Clinical Trials

Today's Date_____

Do you think your dog felt different on this medication?

☐ Yes ☐ No

[If yes:]
Please describe the changes you observed in your pet:

Enter free-response here

Do you think your dog's mobility improved on this medication?

☐ Yes ☐ No

How difficult was this administration method?

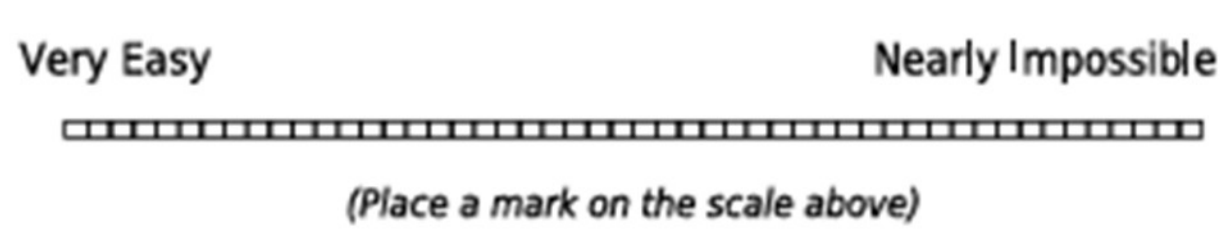

If this product was commercially available for long-term use, would you continue using it?

☐ Yes ☐ No

Please elaborate why you would or would not continue to give this product:

Enter free-response here

References:

1. Harris PA, Taylor R, Minor BL, Elliott V, Fernandez M, O'Neal L, et al. The REDCap consortium: Building an international community of software platform partners. Journal of Biomedical Informatics. 2019;95:103208.

2. Paul A. Harris RT, Robert Thielke, Jonathon Payne, Nathaniel Gonzales, Jose G. Conde. Research electronic data capture (REDCap)--a metadata-driven methodology and workflow process for providing translational research informatics support - PubMed. Journal of biomedical informatics. 2009 Apr;42(2).
