# Supplementary material for: Limitations of transmucosal ketamine administration for chronic pain in dogs with osteoarthritis
Source: Front Vet Sci. 2026 Oct 29;13:1934484. doi: 10.3389/fvets.2026.1934484 (PMC13754547; doi:10.3389/fvets.2026.1934484)
Supplement: Supplementary file 4 [file Data_Sheet_4.PDF]

Transmucosal Ketamine Clinical Trial- Full Patient Data

Provided in this document are supplementary data sets for patient outcome measures.

Contents

Transmucosal Ketamine Clinical Trial- Full Patient Data 1
- Objective Gait Analysis 2
  - Percent Body Weight Distribution by Visit: 2
  - OGA Mean %BWD Pre and Post Treatment 3
- Plasma Analysis of Ketamine and Norketamine 4
- Accelerometry: 5
- Canine Brief Pain Inventory (CBPI) 7
- Client Specific Outcome Measures (CSOM) 7
- Owner Administration Logs 7
- End of Trial Survey Comments: 8

Objective Gait Analysis

Percent Body Weight Distribution by Visit:

		Mean %BWD enrollment limb	Post TMK OGA	Post TMK Diff
1	Day 0	14.29		
1	Day 10	14.87	14.31	-0.56
1	Day 17	10.29		
1	Day 24	12.45		
2	Day 0	21.38		
2	Day 10	24.68	22.88	-1.80
2	Day 17	21.64		
2	Day 24	26.72		
3	Day 0	29.07		
3	Day 10	25.02	25.89	0.87
3	Day 17	23.45		
3	Day 24	25.06		
4	Day 0	22.8		
4	Day 10	21.03	23.34	2.31
4	Day 17	20.73		
4	Day 24	21.15		
5	Day 0	29.89		
5	Day 10	34.2	33.6	-0.60
5	Day 17	37.14		
5	Day 24	35.81		
6	Day 0	15.9		
6	Day 10	17.63	15.68	-1.95
6	Day 17	18.33		
6	Day 24	15.67		
7	Day 0	21.05		
7	Day 10	23.45	24.51	1.06
7	Day 17	23.67		
7	Day 24	17.99		
8	Day 0	30.18		
8	Day 10	28.18	x	x
8	Day 17	29.13		
8	Day 24	28.01		
9	Day 0	35.86		

9	Day 10	33.43	x	x
9	Day 17	32.07		
9	Day 24	27.92		

This table demonstrates the mean %BWD for the enrollment limb of each patient. The mean was calculated from the valid OGA trials collected at each visit. The second column includes the available mean %BWD for the affected limb 1-hour post TMK administration for patients who were able to perform valid gait trails on Day 10

OGA Mean %BWD Pre and Post Treatment

Patient	Mean %BWD pre	Mean %BWD Post	Difference
1	14.58	11.37	3.21
2	12.58	16.915	-4.335
3	11.37	23.03	-11.66
4	16.915	23.16	-6.245
5	23.03	24.18	-1.15
6	23.16	27.895	-4.735
7	24.18	27.045	-2.865
8	27.895	24.235	3.66

Mean %BWD Pre +SD	Mean %BWD Post +SD	Pre to post %BWD difference
24.606 $^{+/-}$6.7	23.735 $^{+/-}$ 7.4	-2.370 $^{+/-}$ 5.08

Plasma Analysis of Ketamine and Norketamine

Sample ID	Ketamine concentration (ng/mL)	Norketamine concentration (ng/mL)
A0	0.392	< 0
A30	2.9	143
A60	1.29	148
B0	0.378	< 0
B30	6.21	55.4
B60	2.67	86.4
C0	0.346	< 0
C30	6.46	44.6
C60	1.35	14.4
D0	0.368	< 0
D30	1.83	58.5
D60	0.75	71.7
E0	0.323	< 0
E30	3.01	35.3
E60	2.31	139
F0	0.651	< 0
F30	8.23	26.2
F60	1.23	19.1
G0	0.384	< 0
G30	2.39	29.6
G60	1.91	37
H0	0.409	< 0
H30	9.87	31.2
H60	4.19	75.5

Caption for Table 1: Results of plasma liquid-chromatography. Patients are labeled alphabetically with “A” corresponding to patient #1 through “H” corresponding to patient #8. Data is listed in chronological order, with Time 0 (immediately prior to administration), 30-minutes post administration, and 60-minutes post administration. *Values ≤1.0 in this assay are equivalent to a concentration of 0ng/ml based on the calibration of the lower limit of quantification (LLOQ) of the assay.

Mean Plasma Concentrations for Figure 2:

**Ketamine**	**Mean**	**SD**	**Norketamine**	**Mean**	**SD**
Time-0	*0.361	*0.102	Time-0	0.000	0.00
Time-30	4.544	2.997	Time-30	47.089	28.286
Time-60	1.744	1.222	Time-60	65.678	46.685

Using the values described in **Table 1,** mean concentration and standard deviation were calculated and represented in the graph for **Figure 2.** *Values ≤1.0 in this assay are equivalent to a concentration of 0ng/ml based on the calibration of the lower limit of quantification (LLOQ) of the assay.

Table 1.1

I3	69.8	44.6
I30	1.19	108
I60	0.499	102

Table 1.1 lists results of plasma liquid-chromatography for patient #9 labeled as "I" in this table had a deviation of protocol due to loss of the sample from time-0. A replacement sample was collected immediately after TMK administration, with clinical notes identifying the replacement sample was acquired approximately 3-minutes after TMK administration. Data is listed in chronological order, with time points at 3-minutes, 30-minutes, and 60-minutes post administration.

Accelerometry:

Baseline			
**Activity Intensity**	**Sedentary**	**Light**	**Vigorous**
**Average**	1290.48	141.51	8.01
**SD**	51.01	48.33	6.25
**SE**	5.38	5.09	0.66
**Post Treatment**			
**Activity Intensity**	**Sedentary**	**Light**	**Vigorous**
**Average**	1302.57	130.98	6.45
**SD**	44.33	41.84	4.68
**SE**	4.27	4.03	0.45
**Difference between Pre- and Post- Treatment**			
**Activity Intensity**	**Sedentary**	**Light**	**Vigorous**
	12.09	-10.53	-1.56

This table demonstrates the time spent in each activity zone in minutes for n=9 dogs. Standard deviation (SD) and standard error (SE) are included.

Subjective Orthopedic Scores (SOS)

SOS pre- and post-treatment

Patient#	1	2	3	4	5	6	7	8	9
Pre	15	16.5	21.5	17.5	16	19.5	14.5	13	18.5
Post	14	15.5	21	16.5	13.5	19	14	13.5	16
Change	1	1	0.5	1	2.5	0.5	0.5	-0.5	2.5

This table demonstrates SOS scores reported as the total sum of individual scores from each visit. The pre-treatment values are the means of the total sums from Day-0 and Day-10, while the post-treatment values are reported as the means of totals from Day-17 and Day-24

Canine Brief Pain Inventory (CBPI)

CBPI scores with PSS and PIS

Patient #	PSS- Day 0	PSS- Day 24	Decrease in score:	PIS- Day 0	PIS- Day 24	Decrease in score:	CBPI- Day 0	CBPI- Day 24	Decrease in score:
*1	21	-		33	-		54	-	
2	26	13	13	46	35	11	72	48	24
3	20	20	0	40	45	-5	60	65	-5
*4	23	-		23	-		46	-	
5	13	9	4	34	20	14	47	29	18
6	22	20	2	32	27	5	54	47	7
7	19	17	2	41	29	12	60	46	14
8	20	14	6	29	16	13	49	30	19
9	15	15	0	25	22	3	40	37	3

Caption for Table 5: Table 5: CBPI scores broken down into PSS and PIS for enrollment (Day-0) and post-treatment (Day-24). *Values are included only for patients with completed surveys from all visits.

Client Specific Outcome Measures (CSOM)

Patient #	Mobility Score Day 0	Mobility Score Day 24	Difference	Behavior Score Day 0	Behavior Score Day 24	Difference
2	20	19	1	13	7	6
3	17	22	-5	3	4	-1
5	18	12	6	4	11	-7
6	20	16	4	5	8	-3
7	13	12	1	6	7	-1
8	20	10	10	5	12	-7
9	15	13	2	6	9	-3

CSOM scores for mobility and behavior comparing enrollment Day-0 and study completion at Day-24. Values are included only for patients who completed all study surveys.

Owner Administration Logs

Total Reported Doses	Success	Chew/Swallow	Spit out/ other	Not noted
334	181	113	30	10

End of Trial Survey Comments:

Patient	Did your pet feel different?	Describe the changes you observed in your pet	Was your dog's mobility improved on this medication?	How difficult was this administration? 0-10*	If this product was commercially available for long-term use, would you continue using it?	Please elaborate:
2	Yes	Increased energy 1-2hrs after dose. Energy lasting for 1hr	Yes	8	No	I would use it if it was a miracle drug. Not enough improvement for the difficulty of administration.
3	Yes	At one point she seemed better, then not so much. I think it is more related to her neurologic coordination than pain.	Yes	5	Yes	mostly difficult to administer
5	Yes	I have definitely seen an improvement in [PET]'s stair-climbing. She used to "wind up" before she went up the stairs, like she was gathering energy. She also hopped up the stairs with two legs at a time. Now, she can go up stairs using opposite legs.<br>[PET]'s limp seems less pronounced and she has an easier time getting up from her bed after a nap.<br>She seemed to have more energy in the morning, especially the hour after she had her ketamine. She wants to play at that time, which she did not do before.<br>My spouse said he thinks [PET] has been slightly calmer, also.	Yes	8	Yes	I will say yes/maybe, depending on the format of the ketamine. If it were to be on the market as is, I doubt we would continue it. [PET] did not like either the taste or texture and frequently spat out the cube unless I could completely mask it. I had a few times that I was successful in putting the cube in her cheek and the cube dissolved, and a few when I put the cube in her cheek and she was able to get to it with her tongue and spat it out, and a few times when I dropped the dose because I could not keep it in my fingers until it melted.
6	Yes	he was definitely sleepier for first 30 or so minutes after getting a dose.	Yes	6	No	I think the administration is not ideal. [PET] is relatively easy going and I still had trouble ~50% of the time when giving it. If it was more of a gel so that the application went quicker then I'd definitely consider doing it long term to see if I see better improvement of his mobility!
7	Yes	He was more sedate than usual and had some dissociation	No	6	No	It was difficult to give this medication every 8 hours. If it was every 12 hours it would be easier to do. I also did not like how sedate it made [PET] as he did not seem to be himself as much

						at least the first 2-3 hrs post administration
8	Yes	Slight sedation	No	1	Yes	I would use it when she has bad pain days but not every day.
9	Yes	After the first week of ketamine, she was much brighter and active. She jumped in the car which she hadn't done in at least a year. She was running around the yard.	Yes	8	No	Only because of the difficulty in administration. If it was available in some other transmucosal form, I definitely would give it.

*How difficult was administration 0-10

0= Very Easy 10= Nearly Impossible
